# Legal Professionals' Knowledge of Eyewitness Testimony in China: A Cross-Sectional Survey

**DOI:** 10.1371/journal.pone.0148116

**Published:** 2016-02-01

**Authors:** Lina Jiang, Dahua Luo

**Affiliations:** 1 College of Humanities and Law, Hangzhou Dianzi University, Hangzhou, PRC; 2 Institute of Evidence Law and Forensic Science, China University of Politics Science and Law, Beijing, PRC; Xi'an Jiaotong University School of Medicine, CHINA

## Abstract

**Purpose:**

To examine legal professionals’ knowledge of a wide range of factors that affect eyewitness accuracy in China.

**Methods:**

A total of 812 participants, including 210 judges, 244 prosecutors, 202 police officers, and 156 defense attorneys, were asked to respond to 12 statements about eyewitness testimony and 3 basic demographic questions (i.e., gender, age, and prior experience).

**Results:**

Although the judges and the defense attorneys had a somewhat higher number of correct responses than the other two groups, all groups showed limited knowledge of eyewitness testimony. In addition, the participants’ responses to only four items (i.e., weapon focus, attitude and expectations, child suggestibility, and the impact of stress) were roughly unanimous within the four legal professional groups. Legal professionals’ gender showed no significant correlations with their knowledge of eyewitness testimony. Prior experiences were significantly and negatively correlated with the item on the knowledge of forgetting curve among judges but positively correlated with two items (i.e., attitudes and exposure time) among defense attorneys and with 4 statements (i.e., the knowledge of attitudes and expectations, impact of stress, child witness accuracy, and exposure time) among prosecutors.

**Conclusions:**

The findings suggest that knowledge of the factors that influence eyewitness accuracy must be more effectively communicated to legal professionals in the future.

## Introduction

Eyewitness testimony in a trial is often important as a form of evidence in courts. However, its use has raised various questions about its reliability and validity within court cases. Much evidence has demonstrated that eyewitness error plays a central role in at least half of all wrongful convictions [1–3]. For example, a previous study has shown that 52 of the first 62 DNA-based exoneration cases involved eyewitness testimony [4]. According to the statistics conducted by the innocence project, eyewitness misidentification occurred in 75% of wrongful convictions exoneration by the DNA test in the United States of America [4–6]. Similarly, Liu and Guo (2009) found that 39 of 137 wrongful convictions in China resulted from eyewitness errors [7].

Psychologists have conducted a substantial amount of research on the limitation of eyewitness testimony, and they have demonstrated that eyewitness evidence is not as reliable as popular opinion or as the courts believe. Thus, considering the important roles legal professionals play in the collection and evaluation of eyewitness testimony [8], it is obviously important to educate them about eyewitness testimony. For example, police officers are in charge of collecting evidence from eyewitnesses and taking official reports. Sometimes, US judges evaluate whether eyewitness testimonies are contaminated by memory-distorting factors. Jurors are also asked by the judges to consider these factors when they evaluate the accuracy of eyewitness testimony [9].

To date, many studies have assessed the knowledge of legal professionals, such as judges [10–12], prosecutors [3, 13], defense attorneys [13, 14], and police officers [13, 15], about eyewitness testimony. These findings have suggested that the majority of legal professionals were not familiar with the factors that influenced the accuracy of eyewitness testimony. For example, Magnusson and colleagues (2010) have shown that both male and female legal professionals had limited knowledge of eyewitness testimony [5]. Kebbell and Milne (1998) found that police officers were similar to lay people in terms of their knowledge of eyewitness testimony [15]. Similarly, Wise and Safer (2010) found that U.S. judges had no more knowledge than undergraduates about those factors of the accuracy of eyewitness testimony, but both groups were less knowledgeable than law students [16].

These available studies have provided some important information about legal professionals’ knowledge of eyewitness testimony. However, little attention has been paid to legal professionals from non-Western countries. To our knowledge, only one study has compared the differences between knowledge of eyewitness testimony among judges in China and America; the study reported that compared with US judges, Chinese judges had less knowledge of the factors that might affect the reliability of eyewitness testimony [12]. As the largest legal system in the world, the Chinese legal system is a complex mix of traditional Chinese approaches and Western influences. For example, in China, judges have less direct contact with witnesses because they usually provide written testimony instead of appearing in court. Chinese prosecutors have a series of powers, including prosecution, investigation, and supervision of the whole judicial procedure. Additionally, defense attorneys in China have relatively less freedom to meet with the defendants and obtain information from the witnesses [17]. These unique characteristics suggest that it is imperative to examine legal professionals’ knowledge of the accuracy of eyewitness testimony in China.

Based on the available literature, the current study used 12 eyewitness statements from previous studies [18–20], and it examined Chinese legal professionals’ (i.e., judges, prosecutors, police officers, and defense attorneys) knowledge of eyewitness testimony. Specifically, we formed the following hypotheses: 1) Chinese legal professionals had limited knowledge of eyewitness testimony; 2) there was a low level of consensus within the group of Chinese legal professionals; 3) there were no between-group differences among legal professionals in terms of their knowledge of eyewitness testimony; and 4) the gender and prior experiences of the legal professionals would not be significantly correlated with their knowledge of eyewitness testimony.

## Method

### Participants and procedure

A total of 1,000 questionnaires were mailed to the relevant institutions, and they were distributed by liaisons to judges, prosecutors, police officers, and defense attorneys. This study was approved by the directors of the concerned institutions and the Research Ethics Committee of China University of Politics Science and Law. After providing written informed consent, the participants completed separately the confidential questionnaire in their offices. A total of 812 people completed the questionnaire and returned them to the liaisons within the required time period. Of the 812 participants, 210 were judges (135 males and 75 females), ranging in age from 23 to 60 years (mean = 39.1 years, *SD* = 7.1 years); 244 participants were prosecutors (195 males and 49 females), ranging in age from 23 to 60 years (mean = 41.4 years, *SD* = 7.1 years); 202 participants (195 males and 7 females) were police officers, ranging in age from 23 to 52 years (mean = 36.9 years, *SD* = 3.8 years); and 156 participants were defense attorneys (104 males and 52 females), ranging in age from 22 to 60 years (mean = 32.7 years, *SD* = 6.5 years).

### Materials

The questionnaire included 12 statements about eyewitness testimony and 3 basic demographic questions (i.e., gender, age, and prior experience). In the current study, prior experience was measured by working time. The 12 statements about eyewitness testimony consisted of 9 items (items 1–9 in Table 1) from the Kassin, Tubb, Hosch, and Memon (2001) study [18], 2 items (items 10–11 in Table 1) from the Wise and Safer (2004) study [19]; and 1 item (item 12 in Table 1) from the Deffenbacher et al. (2004) study [20]. The items were chosen based on their potential effects on the accuracy of eyewitness testimony [19] and their implication in Chinese judicial practice. All items were translated from English to Chinese and back-translated by a bilingual team of professionals. Participants responded to the items on a 4-point scale from 1 (strongly disagree) to 4 (strongly agree). In current study, to calculate the percent correct, we combined the responses of “agree” and “strongly agree” as a correct answer and the responses of “disagree” and “strongly disagree” as a false answer.

**Table 1:** Eyewitness items and statements.

Items	Statements
1. Weapon focus*	The presence of a weapon can impair an eyewitness’s ability to accurately identify the perpetrator’s face.
2. Exposure time*	The less time an eyewitness has to observe an event, the less well he or she will remember it.
3. Forgetting curve*	The rate of memory loss for an event is greatest right after the event, and then it levels off over time.
4. Accuracy/confidence*	The confidence of an eyewitness is not a good predictor of his or her accuracy in identifying the defendant as the perpetrator of the crime.
5. Unconscious transference*	Eyewitnesses sometime identify a culprit as someone they have seen in another situation or context.
6. Attitudes and expectations*	An eyewitness’s perception and memory of an event may be affected by his or her attitudes and expectations.
7. Child witness accuracy	Young children are less accurate as witnesses than adults.
8. Child suggestibility*	Young children are more vulnerable than adults to interviewer suggestion, peer pressures, and other social influences.
9. Conducting lineups*	A police officer who knows which member of the lineup or photo array is the suspect should not conduct the lineup or photo array.
10. Effects of post-event information*	Eyewitness testimony about an event often reflects not only what a witness actually saw but also information obtained later from other witnesses, police, media, etc.
11. Line-up presentation format*	Witnesses are more likely to misidentify someone in a culprit-absent lineup when it is presented in a simultaneous procedure (i.e., all members of a lineup are present at the same time) as opposed to a sequential procedure (i.e., all members of a lineup are presented individually).
12. Impact of stress*	Very high stress has a negative effect on the accuracy of testimony.

*Note*. With the answer that is most likely to be the correct answer according to current memory science, indicated by an asterisk.

## Results

### Legal professionals’ knowledge of eyewitness testimony

Based on the findings from Kassin et al. (2001) and other previous studies [20–23], we estimated the rates of correct responses to the 12 statements among four groups of legal professionals’ (i.e., judges, prosecutors, police officers, and defense attorneys). As shown in Table 2, the correct rates ranged from 40% to 77% (average, 58%) for judges, from 42% to 76% (average, 57%) for the prosecutors, from 46% to 75% (average, 57%) for the police officers, and from 44% to 79% (average, 61%) for the defense attorneys. In addition, as shown in Table 2, half of the correct rates exceeded the level of chance (50%) for all four groups.

**Table 2:** The percentage of judges, prosecutors, police officers and defense attorneys who responded correctly.

Items	Judges (*n* = 210)	Prosecutors (*n* = 244)	Police officers (*n* = 202)	Defense attorneys (*n* = 156)
1. Weapon focus	76%***	76%***	75%***	75%***
2. Exposure time	43%	57%*	50%	58%*
3. Forgetting curve	67%***	70%***	65%***	60%*
4. Accuracy/confidence	42%*	46%	54%	44%
5. Unconscious transference	57%	54%	62%***	50%
6. Attitudes and expectations	77%***	60%**	60%*	76%***
7. Child witness accuracy	52%	45%	51%	58%
8. Child suggestibility	75%***	67%***	64%***	78%***
9. Conducting lineups	50%	48%	52%	49%
10. Effects of post-event information	61%***	54%	62%***	50%
11. Lineup presentation format	40%**	42%*	46%	51%
12. Impact of stress	59%**	59%**	57%*	79%***

*Note*.

* *p* < .05

** *p* < .01

*** *p* < .001

Student’s t-test compared to chance value χ = 50%.

### Within-group comparisons

Benton et al. (2006) have used the criterion of 75% as being suggestive of a unanimous answer [10]. According to this criterion, the participants’ responses to the four items (i.e., weapon focus, attitude and expectations, child suggestibility, and the impact of stress) were roughly unanimous within the four groups of legal professionals. Specifically, for *weapon focus*, the majority of legal professionals (i.e., 76% of judges, 76% of prosecutors, 75% of police officers, and 75% of defense attorneys) believed that the presence of a weapon might impair an eyewitness’s ability to accurately identify a perpetrator’s face. For *attitude and expectations*, 77% of judges and 76% of defense attorneys proposed that an eyewitness’s perception and memory for an event might be affected by his or her attitudes and expectations. Additionally, for *child suggestibility*, 75% of judges and 78% of defense attorneys believed that children (as witnesses) were more likely influenced by an interviewer’s suggestion, peer pressures, and other social influences. Finally, for *the impact of stress*, 79% of defense attorneys thought that very high stress at the time of the observation might affect the accuracy of eyewitness testimony.

### Between-group comparisons

Analysis of variance was conducted for the knowledge of the 12 statements between the four professional groups (i.e., judges, prosecutors, police officers, and defense attorneys). The results showed that there were significant effects between the four legal professional groups for the following 4 items: attitudes and expectations (*F*_(3, 808)_ = 9.12, *p* < .001), the impact of stress (*F*_(3, 808)_ = 17.11, *p* < .001), exposure time (*F*_(3, 808)_ = 2.75, *p* < .05), and accuracy-confidence (*F*_(3, 808)_ = 3.08, *p* < .05). Specifically, for *attitudes and expectations*, the judges (Mean = 2.92, *SD* = .75) reported higher scores of attitude and expectations than the police officers (Mean = 2.67, *SD* = .95) and prosecutors (Mean = 2.70, *SD* = .84). The defense attorneys (Mean = 3.06, *SD* = .81) had higher attitude and expectations scores than the police officers and prosecutors. For *impact of stress*, the defense attorneys (Mean = 3.15, *SD* = .76) reported higher impact of stress scores than the judges (Mean = 2.62, *SD* = .76), prosecutors (Mean = 2.63, *SD* = .79), and police officers (Mean = 2.65, *SD* = .93). For *exposure time*, the judges reported lower scores (Mean = 2.45, *SD* = .81) than the prosecutors (Mean = 2.61, *SD* = .81) and defense attorneys (Mean = 2.65, *SD* = .66). The police officers had lower scores than the defense attorneys. For *confidence*, the judges (Mean = 2.27, *SD* = .89) demonstrated lower accuracy-confidence scores than the police officers (Mean = 2.45, *SD* = .91) and defense attorneys (Mean = 2.53, *SD* = .74).

### Correlations between legal professionals’ knowledge of eyewitness testimony and gender and prior experiences

To examine the relationships between knowledge of eyewitness and gender and prior experiences within the four different legal professional groups, we conducted a series of correlations analyses. The results showed that among the legal professionals, gender had no significant correlations with their knowledge of eyewitness testimony. Additionally, judges’ prior experiences were significantly and negatively correlated with the knowledge of forgetting curve (*r* = -.14, *r*^2^ = .02, *p* < .05). Prosecutors’ prior experiences were significantly and positively correlated with the knowledge of attitudes and expectations (*r* = .27, *r*^2^ = .08, *p* < .01), impact of stress (*r* = .21, *r*^2^ = .04, *p* < .01), child witness accuracy (*r* = .20, *r*^2^ = .04, *p* < .01), and exposure time (*r* = .15, *r*^2^ = .02, *p* < .05). In addition, defense attorneys’ prior experiences were found to be significantly and positively correlated with the knowledge of attitudes (*r* = .22, *r*^2^ = 05, *p* < .01) and exposure time (*r* = .17, *r*^2^ = .03, *p* < .01). Finally, police officers’ prior experiences were not significantly correlated with the knowledge of eyewitness testimony.

## Discussion

The current study examined legal professionals’ knowledge of eyewitness testimony in China. Several important findings emerged. First, similar to previous studies [3, 10, 11, 18, 19, 24], the results of the current study showed that the proportions of correct responses to the 12 statements were 58% for the judges, 57% for the prosecutors, 57% for the police officers, and 61% for the defense attorneys. These findings suggest that legal professionals showed limited knowledge of the limitations of memory and the factors known to influence eyewitness reliability. One possible explanation for these results is that in judicial practice, it is difficult for legal professionals to receive immediate feedback (eyewitness error is usually found some years later) from their evaluation of eyewitness testimony, which gives them less opportunity to improve their knowledge of eyewitness testimony [25]. Furthermore, in China, the training and education that legal professionals receive do not usually involve the reliability of eyewitness testimony.

Second, the results of the present study showed that the majority of 12 items did not reach the level of consensus within each group (i.e., judges, prosecutors, police officers and defense attorneys). In this case, when entering the legal system, citizens might be treated unequally due to the different evaluations of legal professionals on eyewitness testimony. Thus, it is necessary to develop educational programs that target legal professionals, who are involve in all phases of the legal process [26].

Furthermore, the results of the current study found that there were significant effects between the four legal professional groups for the following 4 items (i.e., attitudes and expectations, the impact of stress, exposure time, and accuracy-confidence). One possible explanation for this result is that the four legal professionals (i.e., judges, prosecutors, police officers and defense attorneys) play different roles in the legal process [8, 27]. For example, during the trial, the judges must evaluate the accuracy of eyewitness testimony to make their final decisions. Chinese prosecutors must investigate and supervise the entire judicial procedure. Defense attorneys should improve the awareness of eyewitness errors to obtain and win more cases.

Additionally, the findings suggested that legal professionals’ knowledge of eyewitness testimony did not vary across gender. In addition, consistent with previous studies [11, 19], the results of the current study showed that among three of legal professionals groups (i.e., judges, defense attorneys, and police officers), prior experiences were significantly correlated with few statements, in terms of the knowledge of eyewitness testimony. This finding suggests that having served as a judge/police officer/ defense attorney for a longer time does not enhance the understanding of eyewitness testimony. One potential explanation is that after the eyewitness testimony was evaluated [16], the judges and police officers did not receive any immediate feedback on whether the eyewitness made accurate and inaccurate testimony. Furthermore, in Chinese judicial practice, witnesses seldom appear in court, thus, judges have brief, infrequent, and superficial exposure to eyewitness materials [12].

On the contrary, the results of the current study showed that prior experiences were significantly and positively correlated with the 4 statements (i.e., the knowledge of attitudes and expectations, impact of stress, child witness accuracy, and exposure time) among prosecutors. These findings suggested that prosecutors’ knowledge of testimony might be influenced by their prior experiences. In China, prosecutors have a series of powers, including investigating crimes, approving arrests, representing the government during trials, supervising the legality of court proceedings and the implementation of court decisions, all of which result in experienced prosecutors being more inclined to focus on the accuracy of eyewitness testimony.

## Limitations

Several limitations of the current study should be mentioned. First, in the current study, we evaluated the accuracy of the participant responses based on the findings from a previous study [10]. However, the findings will be revised based on future published research. Thus, caution is needed when interpreting our results and comparing them to studies with similar measures. Second, several important factors (e.g., minor details, mugshot-induced bias, and effects of a hat) were not included in the present study. Future study should further examine legal professionals’ knowledge of these factors. Finally, participants in the current study came from the five cities (i.e., Beijing, Guangzhou, Jinan, Chengdu, and Zhengzhou) in China. Thus, it is unknown whether the findings of the present study could be generalized to other samples of legal professionals.

## Conclusion

Despite these limitations, the current study provided some interesting insights into Chinese legal professionals’ knowledge of the limitation of human memory and other factors that may be distorted by the processes. Specifically, the Defense attorneys had the best knowledge of eyewitness testimony among the four groups. Moreover, legal professionals’ knowledge of eyewitness testimony often did not reach the level of consensus within the same groups. In addition, the differences between the legal professional groups were only found in the four items (i.e., attitudes and expectations, the impact of stress, exposure time, and accuracy-confidence). Finally, in general, legal professionals’ gender was not significantly correlated with their knowledge of eyewitness testimony. But Prosecutors’ prior experiences were positively related to the knowledge of attitudes and expectations, impact of stress, child witness accuracy, and exposure time. These findings suggest that knowledge of the factors that influence eyewitness accuracy must be more effectively communicated to legal professionals in the future.
